# Imaging the rotational mobility of carbon dot-gold nanoparticle conjugates using frequency domain wide-field time-resolved fluorescence anisotropy

**DOI:** 10.1117/1.JBO.28.5.056001

**Published:** 2023-05-23

**Authors:** Gilad Yahav, Shweta Pawar, Yitzchak Weber, Bar Atuar, Hamootal Duadi, Dror Fixler

**Affiliations:** Bar Ilan University, The Faculty of Engineering and the Institute of Nanotechnology and Advanced Materials, Ramat Gan, Israel

**Keywords:** fluorescent lifetime, rotational correlation time, fluorescence lifetime imaging microscopy, frequency domain, fluorescence anisotropy, fluorescence anisotropy decay

## Abstract

**Significance:**

Wide-field measurements of time-resolved fluorescence anisotropy (TR-FA) provide pixel-by-pixel information about the rotational mobility of fluorophores, reflecting changes in the local microviscosity and other factors influencing the fluorophore’s diffusional motion. These features offer promising potential in many research fields, including cellular imaging and biochemical sensing, as demonstrated by previous works. Nevertheless, θ imaging is still rarely investigated in general and in carbon dots (CDs) in particular.

**Aim:**

To extend existing frequency domain (FD) fluorescence lifetime (FLT) imaging microscopy (FLIM) to FD TR-FA imaging (TR-FAIM), which produces visual maps of the FLT and θ, together with the steady-state images of fluorescence intensity (FI) and FA (r).

**Approach:**

The proof of concept of the combined FD FLIM/ FD TR-FAIM was validated on seven fluorescein solutions with increasing viscosities and was applied for comprehensive study of two types of CD-gold nano conjugates.

**Results:**

The FLT of fluorescein samples was found to decrease from 4.01±0.01 to 3.56±0.02  ns, whereas both r and θ were significantly increased from 0.053±0.012 to 0.252±0.003 and 0.15±0.05 to 11.25±1.87  ns, respectively. In addition, the attachment of gold to the two CDs resulted in an increase in the FI due to metal-enhanced fluorescence. Moreover, it resulted in an increase of r from 0.100±0.011 to 0.150±0.013 and θ from 0.98±0.13 to 1.65±0.20  ns for the first CDs and from 0.280±0.008 to 0.310±0.004 and 5.55±1.08 to 7.95±0.97  ns for the second CDs. These trends are due to the size increase of the CDs-gold compared to CDs alone. The FLT presented relatively modest changes in CDs.

**Conclusions:**

Through the combined FD FLIM/ FD TR-FAIM, a large variety of information can be probed (FI, FLT, r, and θ). Nevertheless, θ was the most beneficial, either by probing the spatial changes in viscosity or by evident variations in the peak and full width half maximum.

## 1 Introduction

Fluorescence lifetime (FLT) imaging microscopy (FLIM) probes changes in the excited state kinetics of intrinsic or extrinsic fluorophores, which are sensitive to their photophysical properties and the chemical and physical nature of the molecular environment of the labeled macromolecules. Therefore, FLIM is popular for investigating biochemical reactions and molecular interactions within the cellular environment that are valuable for biological and medical imaging.^1^^,^^2^ These include using Förster resonance energy transfer (FRET) to study both protein interactions and conformational changes as well as using fluorescent molecular rotors to map the viscosity (yet, generally the FLT is independent of solvent viscosity).^3^^,^^4^ Furthermore, FLIM is utilized for mapping the temperature,^5^ pH,^6^ refractive index,^7^ intracellular molecules (e.g., calcium, magnesium, chromatin, myoglobin, and antigens),^1^^,^^3^ glucose,^8^ and oxygenation.^9^ FLIM was also used for the identification of chromosomal abnormalities in Leukemias,^10^ carriers of BRCA mutations^11^ and detection of metastatic cells in the cerebrospinal fluid of children with medulloblastoma.^12^ Other applications include quantification of the metabolic state in a cell model of Parkinson’s disease,^13^ tracking apoptosis and stimulation in individual cells,^14^ and studying of fluorophore-conjugated gold nanoparticles (AuNPs) or nanorods in phantoms.^15^

If time-resolved fluorescence anisotropy (TR-FA) analysis is integrated with FLIM, information about the rotational mobility of the fluorophore and its conjugated biomolecule can effectively be extracted. Changes in the rotational mobility arise readily in cell biology from variations in local microviscosity or other constraints to the diffusional motion, such as changes in the size, shape, flexibility, and associative behavior of fluorophore-biomolecule constructs. This can provide significant insight into the corresponding biomolecular carrier within a cell.^16^^,^^17^

For spherical rotors, the rotational diffusion of the fluorophore is typically characterized by the rotational correlation time, θ, which can be calculated using the Debye-Stokes-Einstein hydrodynamic model^4^

$$\theta =\frac{\eta V}{\mathrm{RT}},$$ (1)

where V is the apparent molecular volume of the rotating molecule, η refers to the viscosity of the solvent, R is the universal gas constant, and T is the temperature. The linear dependence of θ on viscosity can be exploited to quantify the viscosity itself and obtain a two-dimensional viscosity map.

Single point steady-state and TR-FA measurements are well established and routinely used for various applications in many laboratories. These include studying the dynamic properties of proteins,^18^^–^^20^ estimating the internal viscosities of membranes,^21^ performing fluorescence-polarization immunoassays,^22^^,^^23^ probing molecular proximity manifested by hetero- or homo-FRET assays,^20^^,^^24^^,^^25^ and monitoring cellular activities.^14^^,^^26^ Nevertheless, since the pioneering work in rotational correlation time imaging of Siegel et al. in the time domain (TD) (2003)^4^ and Clayton et al. in the frequency domain (FD) (2002),^27^ only few reports described their extension into two-dimensional (2D) TR-FA imaging (TR-FAIM) in the TD^26^^,^^28^^–^^30^ and almost none in the FD.^31^ Nonetheless, there are a variety of works that signify the use of FD measurements for the resolution of fluorescence intensity (FI) and FA decays thus probing cell behavior and monitoring changes in its microenvironment.^16^^,^^32^^–^^37^ Furthermore, the measurement procedures and the data analysis in the FD method are significantly faster, and hence more suitable for clinical applications.^38^ In both FD apparatuses,^27^^,^^31^ the images at different polarizations are acquired using two sets of measurements. However, besides the speed limit (which is crucial for high throughput diagnostic tools), consecutive acquisition is susceptible to inaccuracies that can stem from temperature variations, photobleaching, movement of the sample between the acquisition of the two images and biophysical or biochemical changes among other possibilities.

The great potential in performing TR-FAIM in the FD using simultaneous acquisition of the two polarization component maps directly prompted the present work. Such an integrated system can offer cellular imaging based on maps of both the FLT and the rotational correlation time, which are two intrinsic parameters, and hence relatively insensitive to experimental factors (such as the intensity of the excitation source, detector efficiency, or the fluorophores concentration). Both may vary according to the local chemical and physical environmental factors and across the sample under investigation. Moreover, the dependence of rotational correlation time on the viscosity and dynamic properties of the tissues^4^^,^^27^ can be compared to the dependence of T1 (longitudinal) and T2 (transverse) water or proton relaxation times, which generate the image contrast in magnetic resonance imaging.^16^ This implies that TR-FAIM may become a reliable and powerful diagnostic tool for cellular imaging providing parallel interrogation of several unique features of complex biological structures. TR-FAIM was previously used in biological imaging to study bacteria expressing enhanced green fluorescent protein,^27^ as well as the molecular self-assembly in live cells.^39^ In addition, it was used to differentiate between cancerous and normal prostate tissues areas^40^ and to measure the hydrodynamic radii of anti-VEGF drugs.^41^

Previous studies have illustrated the significant potential of TR-FAIM in various research areas such as cellular imaging and biochemical sensing.^26^^–^^31^ However, to the best of our knowledge, θ imaging has not been explored in carbon dots (CDs).

Since 2004, CDs have attracted great attention and sparked a lot of research.^42^ CDs have substantial benefits over conventional organic dyes and classical quantum dots, including high fluorescence, unique and tunable optical properties, facile synthesis, and superior biocompatibility. These features and more make them preferable candidates for drug administration and bioimaging, photocatalysis, disease detection, and biochemical sensing applications among other research fields.^43^^–^^45^ AuNPs are attractive because of their non-toxic nature, ease of modification, biocompatibility, and adjustable optical properties, as well as their large absorption cross-section.^46^ A nanohybrid created by AuNPs and CDs can retain the optical properties of both the AuNPs and the CDs, it can be employed as a unique nanosized fluorescent material. The near-field interactions of the CDs and the AuNPs can change the fluorescence wavelength making CDs-AuNPs (CDs-Au) constructs suitable FLIM probes. In addition, the increasing of the effective size of the CDs-Au construct relative to the CDs alone makes them favorable for rotational correlation time investigations [Eq. (1)].

We present here a wide-field TR fluorescence imaging system that yields maps of the FI and FLT as well as maps of the steady state FA (r), rotational correlation time (θ), and the fundamental FA ($r_{0}$). This is primarily achieved by incorporating a linear polarizer in the excitation path and polarized beam splitter (PBS) in the emission path of a wide-field FD FLIM system. The latter acquires two polarization-resolved images (polarized parallel and perpendicular to the excitation beam) simultaneously, effectively realizing TR-FAIM. The combined FD FLIM/ TR-FAIM is demonstrated on seven fluorescein solutions with increasing viscosity (achieved by increasing glycerol concentration between 0% and 80%) and is applied to study two type of CDs, distinguishable based on the carbon source, each was examined both with and without a gold attachment.

## 2 Materials and Methods

### 2.1 Optical Setup

**Fig. 1:**
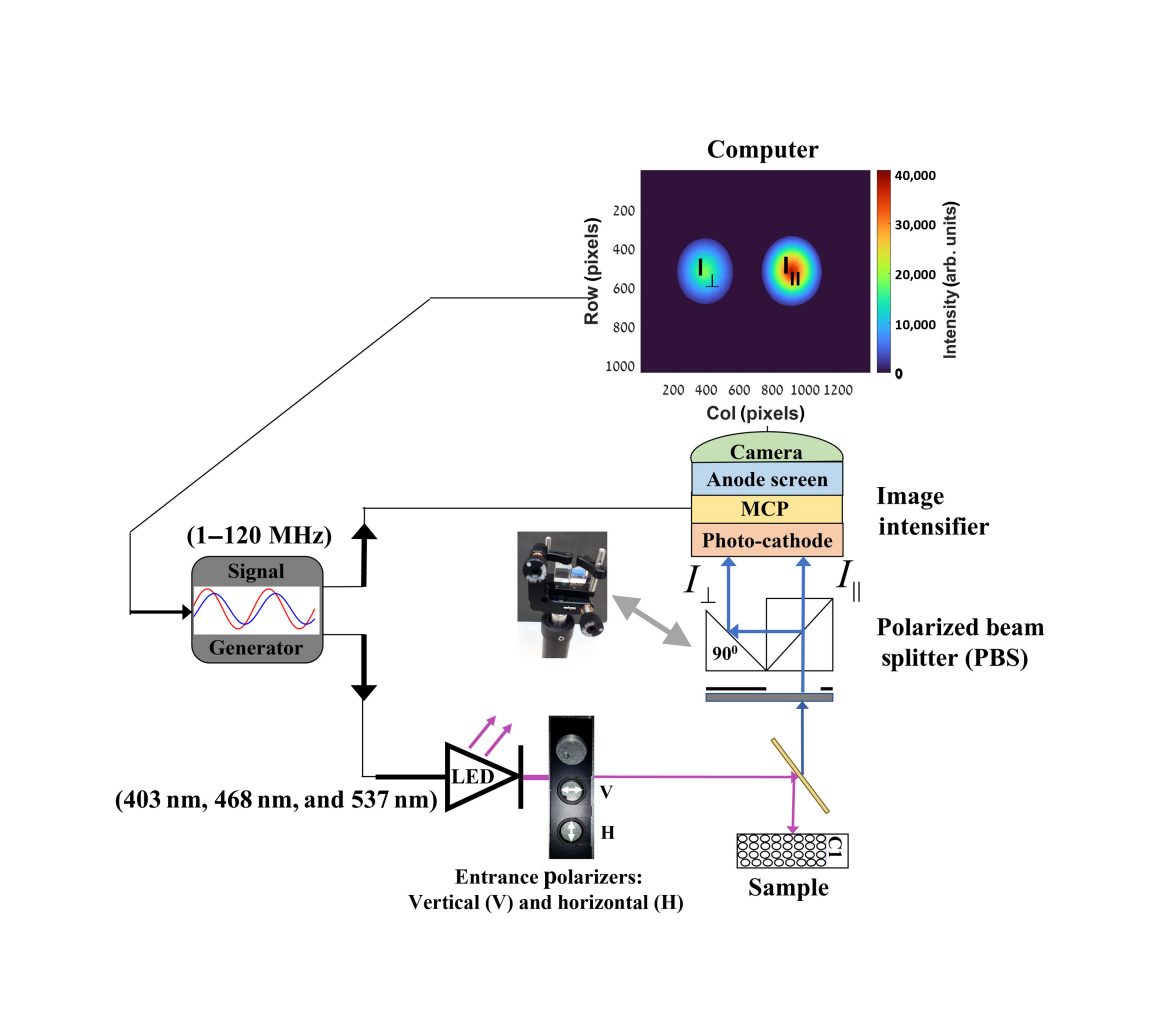
A schematic illustration of the FD TR-FAIM. FA measurements are implemented by adding entrance polarizers (horizontal and vertical) at the output of the multi-LED source and a PBS at the input of the image intensifier of our FD-FLIM system. A pinhole at the input of the PBS adjusts the width of the FI to avoid overlap of the two polarization components of the fluorescence emission. Using a mirror, the two polarized beams, which are extracted by the PBS, arrive in parallel to the CCD camera whose field of view is divided between the two.

The instrumental setup for the FD TR-FAIM, as illustrated in Fig. 1, is based on an existing FD-FLIM technology of Lambert instruments (LIFA, Groningen, The Netherlands).^47^ The correlation time imaging is realized by the addition of fixed linear polarizers (one horizontal and one vertical) in the excitation path and a PBS (Thorlabs Inc., New Jersey, United States) in the emission path as will be explained below. The PBS separates the fluorescence emission into two orthogonal polarizations that are focused on different regions of the charge-coupled device (CCD) camera and consequently allows simultaneous recording of the parallel and perpendicular images.

The excitation source, a multi-light emitting diode (LED) with three different usable wavelengths of 403, 468, (used in this work) and 537 nm, is modulated in a sinusoidal fashion by a signal generator (Prior OptiScan I, Rockland, Massachusetts, United States). This sine wave (AC modulation superimposed upon DC) is characterized by a high frequency in a range between 1 and 120 MHz.^47^ In this research, six modulated frequencies between 20 and 45 MHz, linearly spaced, were used for all samples.

A dragged device containing three windows is placed at the output of the LED to excite the sample with one of three options: a vertical polarizer modulated light (for FA measurements), a horizontal polarizer modulated light (for supporting G factor calculation as will be elaborated in Sec. 2.2.2) and unpolarized modulation light (for calibration purposes).

An Olympus IX-81 inverted microscope with a 10×, NA=0.4 objective (OLYMPUS, Tokyo, Japan) is used to focus the sample. Then, a dichroic mirror within the microscope filters the excitation so that only the FI arrives at the PBS that divides the polarized fluorescence emission to the vertical ($I_{\left|\right|}$) and horizontal ($I_{⊥}$) orientations at the input of the image intensifier.

Besides the parallel (∥) and perpendicular (⊥) images of the FI, images of the their modulation depth ($m_{∥}$ and $m_{⊥}$) and phase shift ($\phi_{∥}$ and $\phi_{⊥}$) are also extracted. These frequency response data (FRD) can be acquired using a gain-modulated detection device (image intensifier) operating at the same frequency as the fluorescence emission but with numerous different phase angles between them (known as a homodyne phase-sensitive detection).^47^ The FRD are calculated in each individual pixel of the image through a FLIM software package computer program (LI-FLIM). This program fully controls the settings of the signal generator, LED, image intensifier and camera, and presents the data in 2D presentations (with a spatial resolution of 1392×1040  pixels). The system’s time resolution (both FLT and θ) is ∼80  ps, which is consistent with the manufacturer’s specifications, and the r value is estimated to be around 0.001.

An added pinhole in the input of the PBS is used to control the width of the fluorescence emission, to prevent an overlap between the two polarized beams. The output of the image intensifier is coupled to a 16-bit CCD camera (${\mathrm{LI}}^{2}\mathrm{CAM}$ MD). The field of view (14.4×10.8  mm) is divided between the two polarization components of the emission.

The two polarized emission components (the parallel, $I_{∥}$ and the perpendicular, $I_{⊥}$) depend on the decay constants of the fluorescent material and the modulation frequency (represented by the angular modulation frequency, ω). They are modulated at the same frequency as the excitation, but phase shifted relative to each other; the AC amplitude of the perpendicular component ($I_{⊥}^{\mathrm{AC}}$) leads the AC amplitude of the parallel component ($I_{∥}^{\mathrm{AC}}$) by Δϕ. In addition, $I_{⊥}$ is reduced relative to $I_{∥}$ by the ratio, Λ, which can be calculated by multiplying the modulation depth (m) and DC ratios of the two polarized beams

$$\Lambda_{\omega }=\frac{I_{∥}^{\mathrm{AC}}}{I_{⊥}^{\mathrm{AC}}}=\frac{m_{∥}}{m_{⊥}}\frac{I_{∥}^{\mathrm{DC}}}{I_{⊥}^{\mathrm{DC}}}.$$ (2)

$\Lambda_{\omega }$ is provided to calculate the modulated anisotropy, $r_{\omega }$, using

$$r_{\omega }=\frac{\Lambda_{\omega }-1}{\Lambda_{\omega }+2}.$$ (3)

By setting ω=0, the steady state FA, r, can be extracted using Eq. (3) where $\Lambda_{0}=I_{∥}^{\mathrm{DC}}/I_{⊥}^{\mathrm{DC}}$.

For single exponential FI and FA decays, r can also be extracted by the Jablonski equation, which is the anisotropy analogue of Perrins polarization equation^48^^,^^49^

$$r=\frac{r_{0}}{1+\tau /\theta }.$$ (4)

The basic principles of the FD analysis were previously discussed.^50^

### 2.2 Data Calibration in FD TR-FAIM/ FD FLIM

All measurements were performed at ambient temperature (20°C). Although the configurations of the FD FLIM and the FD TR-FAIM are only somewhat different, the calibration process of each of these configurations is entirely dissimilar. Thus, this section describes separately each of them.

#### 2.2.1 Calibration for FLT measurements

To resolve the FLT images, the FRD of the FI decays were extracted through a separate experiment by replacing the PBS in the emission path with a linear polarizer oriented 54.7 deg from the vertical (magic angle setting). This configuration eliminates any influence of the FA over the obtained FRD of the FI decays and hence avoids the distortion of the FLTs.^51^ In addition, to remove the frequency dependence of the system itself, a reference (a solution with a known FLT value) of a fluorescein dye (the first sample in Sec. 2.4) was used for calibration.

The corrected phase shift is extracted as

$$\phi_{s}^{r}=\phi_{s}^{m}-\phi_{\mathrm{ref}}^{m}+\phi_{\mathrm{ref}}^{r},$$ (5)

where $\Delta \phi_{\mathrm{ref}}^{m}$ and $\Delta \phi_{\mathrm{ref}}^{r}$ are the measured and the real phase shift of the reference solution, respectively. The terms $\Delta \phi_{s}^{m}$ and $\Delta \phi_{s}^{r}$ are similarly described for the sample. In the same way, the corrected modulation depth is extracted as

$$\frac{1}{m_{s}^{r}}=\frac{1}{m_{s}^{m}}\frac{m_{\mathrm{ref}}^{m}}{m_{\mathrm{ref}}^{r}},$$ (6)

where $m_{\mathrm{ref}}^{m}$ and $m_{\mathrm{ref}}^{r}$ are the measured and the real modulation depth of the reference solution, respectively. The terms $m_{s}^{m}$ and $m_{s}^{r}$ are similarly described for the sample.

#### 2.2.2 Calibration for FA measurements

To calculate the correct DC and AC ratios between $I_{∥}$ and $I_{⊥}$ (and hence the correct r and θ maps), any electrical or optical distortion of the polarization components (such as different transmission efficiencies) should be compensated. It is known that in experimental setups based on PBSs and filters (and not monochromators) as in our case, often the compensation factor (known as G factor) is insignificant (G≈1). Nevertheless, unlike the transmitted beam ($I_{∥}$) in the PBS used in our system that is considerably pure (with an extinction ratio>1000:1), the reflected beam ($I_{⊥}$) has a leakage of approximately L=5% of the other polarization component ($I_{∥}$) over the wavelength range from 420 to 680 nm (the procedure for determining the 5% leakage is explained in detail in Appendix A in the Supplementary Material). This leakage can be fixed using the following equation:

$$I_{⊥}^{\mathrm{corr}}=1.02I_{⊥}^{\exp}-\frac{10}{9}LI_{∥}^{\exp}.$$ (7)

The superscripts exp and corr refer to the measured (experiment) and corrected $I_{⊥}$ or $I_{∥}$, respectively. The coefficients 1.02 and 10/9 are derived from the transmission efficiency of the PBS for the transmitted component $I_{∥}^{\exp}\approx \frac{9}{10}I_{∥}^{\text{real}}$ and for the reflected component (PBS and mirror) $I_{⊥}^{\exp}\approx \frac{98}{100}I_{⊥}^{\text{real}}$. The modulated polarized signals can be described as

$$I_{p}=I_{p}^{\mathrm{DC}}[1+m_{p}\;\sin(\omega t-\phi_{p})],$$ (8)

where the subscript p indicated either ∥ or ⊥. Substituting Eq. (8) in Eq. (7) yields that the DC leakage can be compensated by setting $I=I_{\mathrm{DC}}$ in Eq. (7). The AC leakage can be compensated using the following equations:

$$I_{⊥}^{\mathrm{AC},\mathrm{corr}}=\sqrt{\left(I_{1}{)}^{2}+(I_{2}{)}^{2}+2I_{1}I_{2}\;\cos(\Delta \phi^{\exp}\right)},\;\phi_{⊥}^{\mathrm{corr}}=\sin^{-1}(\phi_{⊥}^{\exp}-\frac{I_{2}}{I_{⊥}^{\mathrm{AC},\mathrm{corr}}}\;\sin(\Delta \phi^{\exp})).$$ (9)

where

$$I_{1}=1.02I_{⊥}^{\mathrm{DC},\exp}m_{⊥}^{\exp},\;I_{2}=-\frac{10}{9}LI_{∥}^{\mathrm{DC},\exp}m_{∥}^{\exp},\;\Delta \phi^{\exp}=\phi_{⊥}^{\exp}-\phi_{∥}^{\exp}.$$ (10)

Full derivation of the DC and AC compensation formulas are presented in Appendix A in the Supplementary Material.

Once the leakage is compensated for, the G factor can be extracted. However, one should notice the geometry of the FD TR-FAIM apparatus. In the conventional L-format or T-format configurations, the detection is collected at right angles to the excitation and hence horizontal excitation results in the same signals in the parallel and perpendicular channels. In our FD TR-FAIM configuration, the fluorescence emission is observed along the same axis as the excitation (collinear setup). For this geometry, rotation of the excitation polarizer reverses the signals in the two polarized observation channels. Thus, the G factor of each pixel is extracted as^4^

$$G(x,y)=\sqrt{\frac{I_{\mathrm{VV}}(x,y)}{I_{\mathrm{VH}}(x,y)}\frac{I_{\mathrm{HV}}(x,y)}{I_{\mathrm{HH}}(x,y)}}.$$ (11)

For each intensity component, we use two subscripts to indicate the orientation of the excitation and emission polarizers where the order of the subscripts represents the order in which the light passes through the two polarizers. For example, $I_{\mathrm{VH}}$ corresponds to vertically polarized excitation and horizontally polarized emission. Accordingly, G(x,y) can be acquired by recording two sets of FI images at vertical excitation ($I_{\mathrm{VV}}$ and $I_{\mathrm{VH}}$) and horizontal excitation ($I_{\mathrm{HV}}$ and $I_{\mathrm{HH}}$). Using Eq. (11), the G factor was found 1.04±0.01. In addition, expectedly, excitation with unpolarized light resulted with no phase shift difference between the two polarized components (within experimental error). Thus, no corrections for instrumental effects on the phase shift were required.

### 2.3 Data Processing in FD TR-FAIM

The FRD of the FI and FA decay data were analyzed with MATLAB 2021b software (MathWorks, Massachusetts, United States). Images of FI and FLT were masked by excluding pixels with FI lower than 5% of the maximal pixel value. In the same manner, images of r and θ were masked by eliminating pixels below a threshold of 5% the maximal $I_{∥}^{\mathrm{DC}}$. In this manner, regions with ill-defined data were eliminated. In addition, images of the FA were calculated after pixel registration of the two polarized emission images by finding the center of gravity of each polarized component. The center of gravity of each image can serve to define a circular or square region of interest (although in this research each image was analyzed using its entire shape).

### 2.4 Preparation of Fluorescein-Glycerol (Fl-Gly) Solutions

As a Fl-Gly system is a popular model to study rotational dynamics,^14^^,^^27^ seven fluorescein solutions (50  μM) were analyzed in this research. They were prepared by dissolving fluorescein (Sigma, St. Louis, Missouri, United States) in phosphate-buffered saline (biological industries, Kibbutz Beit Haemek 25115, Israel) solutions having different viscosity (different glycerol in Phosphate Buffered Saline concentrations: 0%, 30%, 40%, 50%, 60%, 70%, and 80%). All samples had a pH value of 7.4.

### 2.5 Carbon Dots

The hydrothermal technique has been demonstrated to be a simple and successful way to synthesize luminous CDs. Surface modification and heteroatom doping have both been used to improve and regulate the fluorescence properties of CDs. Because of its suitable atom radius with C, high electronegativity, and unique electron configuration, nitrogen (N) is the most used doping element in CDs. N has been shown to alter electron density by injecting electrons into CDs, hence altering the internal electrical environment.^52^ Polyethyleneimine (PEI)-modified CDs (PEI-CDs) was one of the most investigated N-doped CDs among them.^53^^,^^54^

#### 2.5.1 Preparation of CDs

In a glass beaker, for CD1, the carbon source was citric acid (CA) (0.0210 g). CA and PEI (3 mL, 5%) were dissolved in 7-mL distilled water. The translucent fluid was transferred to a Teflon-lined autoclave chamber after 3 mins of stirring. The chamber was then sealed and placed in an oven. The color of the solution changed to yellow after 10 hrs of hydrothermal reaction in the oven at 180°C, suggesting the CDs formation. Without any extra separation or purification, the clean and transparent CD solution was used in future experiments. A similar process was used for CD2, with para phenylenediamine (p-PD) as the carbon source.

#### 2.5.2 Preparation of PEG-Coated AuNPs

*In situ* AuNP preparation was accomplished by adding ${\mathrm{HAuCl}}_{4}.3H_{2}O$ to a $\mathrm{PEG}\text{-}{\mathrm{NH}}_{2}$ solution containing tri sodium citrate. Typically, 340-μL PEG and 0.75-mL 1% trisodium citrate were combined in a 50-mL beaker with a magnetic stirrer and heated to 50°C. Then, with vigorous stirring, 19.74 mg of gold precursor (${\mathrm{HAuCl}}_{4}.3H_{2}O$) was added. The solution was then gradually heated to 80°C and agitated until it turned ruby red. PEG-coated AuNPs are formed when the color changes from translucent to ruby red.

#### 2.5.3 Preparation of CDs-Au Nanohybrid

For the EDC-NHS coupling of CDs with AuNPs, 8  μl of 10  mg/ml EDC solution and 16  μl of NHS were combined to with 1 ml of gold colloid. The solution was vortexed and incubated for 30 mins after the addition. The solution was then centrifuged for 5 mins at 3600 RCF. 1-ml PEG-coated AuNPs and 1-ml pure CDs (CD1 and CD2) were combined well, vortexed, and incubated overnight to give CDs-Au nanohybrid.

## 3 Results and Discussion

To confirm the ability of the FD TR-FAIM system to probe variations in the rotational diffusion of fluorophores, we measured the broadly utilized dye fluorescein in seven solvents with increasing viscosities (achieved by increasing the glycerol concentration: 0%, 30%, 40%, 50%, 60%, 70%, and 80%). All 7 solutions were investigated using the main fluorescence characteristics (FI, FLT, r, θ, and $r_{0}$). Once the proof of concept of the FD TR-FAIM was well established, we investigated the same fluorescence characteristics on two CDs distinguished by their carbon sources as well as the effect of connecting each of these CDs to gold.

The results in this section will be presented using images of the FI, FLT, r, and θ as well as their histograms (of the normalized probability distribution). The histograms were characterized using their mode (maximum amplitude) and full width half maximum (FWHM). Unlike the FLT image, which exhibits a Gaussian distribution, and hence the mean of the distribution is equal to the mode, in the skewed image of θ distribution the mean is typically much higher than the mode (nonetheless only the mode and FWHM are focused on this paper). The mode and FWHM definitions with detailed examples for θ distributions (Fig. S1 in the Supplementary Material) are described in Appendix C in the Supplementary Material.

### 3.1 Imaging a Fl-Gly System

The FD FLIM/ TR-FAIM system was used to image the FI, FLT, r, and θ of 7 fluorescein solutions with increasing viscosities. All seven samples exhibited a higher intensity in the parallel component, indicating sufficient viscosity to resolve the depolarization. Using the chi-square analysis, a single exponential fit was found the most suitable for both the FI and the FA decay analyses and hence it was applied for each image pixel, yielding the FLT and θ maps.

**Fig. 2:**
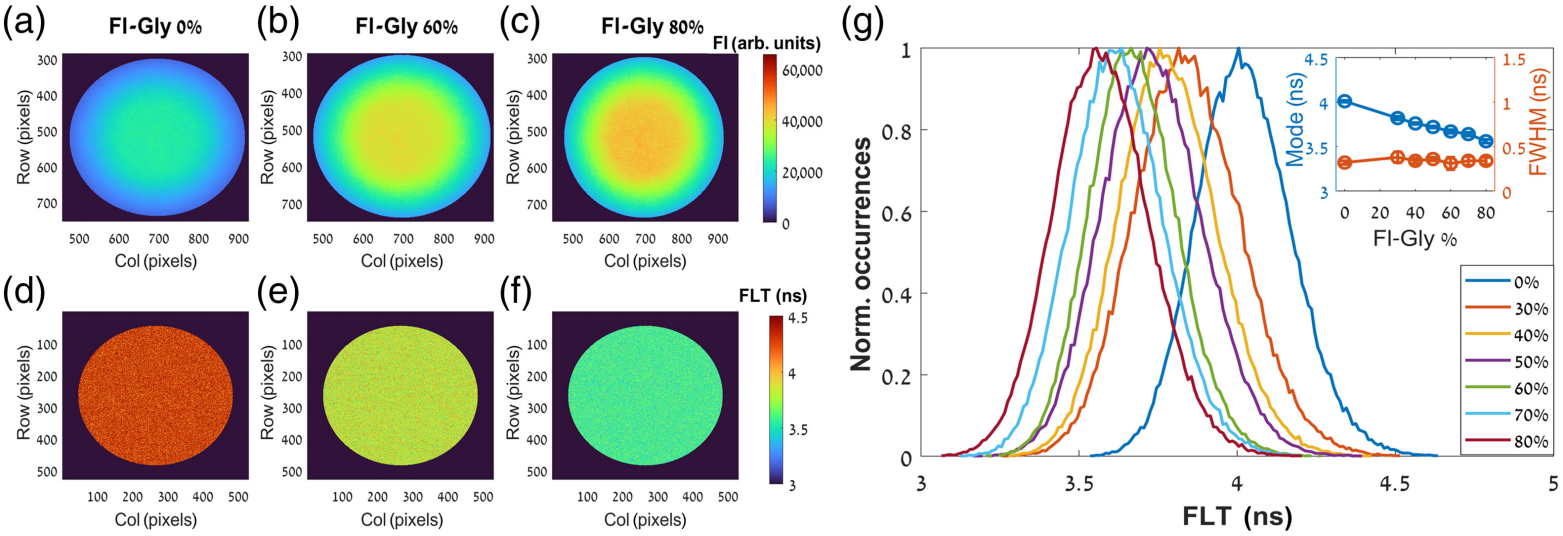
(a)–(c) FI and (d)–(f) FLT maps of Fl-Gly solutions with different glycerol concentrations (0%, 60%, and 80%). The FI and the FLT are represented by a color scale from 0 to 65535 arb. units and 3 to 4.5 ns, respectively. The increasing of glycerol concentration is expressed by the increase of the FI and the decrease of the FLT. The latter is also presented by histograms of the FLT (g) of the seven Fl-Gly solutions (with different glycerol concentrations between 0% and 80%). Inset: the peak of the FLT histograms shows a clear descendant with the increasing of glycerol concentration whereas the FWHM remains constant (blue and orange circles, respectively).

First, the seven Fl-Gly solutions were investigated by the steady-state FI, measured in arbitrary units (arb. units), and the FLT. The findings (shown in Fig. 2 and for FLT also summarized in Table 1) indicate a clear dependence of the FI [Figs. 2(a)–2(c)] and the FLT [Figs. 2(d)–2(f)] on the glycerol concentration (data is presented for example for the 0%, 60%, and 80%). The increase in the glycerol concentration resulted in an increase in the FI and a noticeable decrease in the FLT (from 4.01 ns at 0% to 3.56 ns at 80%). The latter trend is also demonstrated using FLT histograms of the seven solutions [Fig. 2(g)] and observing the peak of each distribution [blue circles in the inset of Fig. 2(g)]. Nevertheless, the FWHM [orange circles in the inset of Fig. 2(g)] of each distribution remains constant.

**Table 1:** Dependence of FI and FA decays parameters on glycerol level (0% to 80%).

Fl-Gly (%)	FLT (ns)		r *		θ (ns)		$r_{0}$
	Mode	FWHM	Mode	FWHM	Mode	FWHM	Mode
**0**	4.01 ± 0.01	0.32 ± 0.02	0.053 ± 0.012	0.036 ± 0.002	0.15 ± 0.05	0.27 ± 0.10	0.40 ± 0.01
**30**	3.82 ± 0.02	0.38 ± 0.06	0.098 ± 0.006	0.042 ± 0.004	0.90 ± 0.13	0.71 ± 0.16	0.38 ± 0.02
**40**	3.76 ± 0.01	0.34 ± 0.03	0.112 ± 0.004	0.040 ± 0.002	1.10 ± 0.16	0.90 ± 0.15	0.37 ± 0.03
**50**	3.72 ± 0.01	0.36 ± 0.02	0.134 ± 0.006	0.043 ± 0.004	2.10 ± 0.26	1.60 ± 0.10	0.36 ± 0.04
**60**	3.67 ± 0.02	0.31 ± 0.07	0.177 ± 0.006	0.044 ± 0.002	4.30 ± 0.34	2.70 ± 0.15	0.33 ± 0.04
**70**	3.64 ± 0.02	0.34 ± 0.05	0.231 ± 0.009	0.045 ± 0.002	7.75 ± 1.30	5.40 ± 0.20	0.31 ± 0.06
**80**	3.56 ± 0.02	0.34 ± 0.04	0.252 ± 0.003	0.044 ± 0.002	11.25 ± 1.87	7.50 ± 0.50	0.30 ± 0.06

* Since typically r has standard deviation <0.01, it is exhibited with an accuracy of 3 decimal place, whereas the FLT, θ, and $r_{0}$ are exhibited with an accuracy of 2 decimal place from the same considerations.

A suspected reason for the increase in the FI with the increasing glycerol concentration could be the prevention of dynamic quenching due to a constraint on rotation at increasing viscosities. As the viscosity increases, the movements of the molecules are more restricted and hence also the collisional quenching.^2^ However, prevention of quenching should also be expressed by FLT increasing^2^ whereas the received trend is the opposite. Viscosity can alter the FLT only in a few fluorophores whose chemical structures enable nonradiative energy relaxation mechanisms through internal twisting (known as molecular rotors). The latter can be hindered with the increase of viscosity and thus can increase the FLT.^2^^,^^55^^,^^56^ Fluorescein is not a molecular rotor, hence this is not the trend in our case.^57^ However, the Strickler–Berg formula (as exhibited in Appendix B in the Supplementary Material) predicts an inverse relation between the FLT of a fluorophore (τ) and the refractive index (n) of its environment ($\tau^{-1}\propto n^{2}$) due to its polarizability.^58^^,^^59^ This is a fundamental relation that applies to all fluorophores, irrespective of their structure or their environment.^60^ Furthermore, the lower degree of contrast in fluorescein FLT is known due to its insensitivity to solvent composition.^61^

**Fig. 3:**
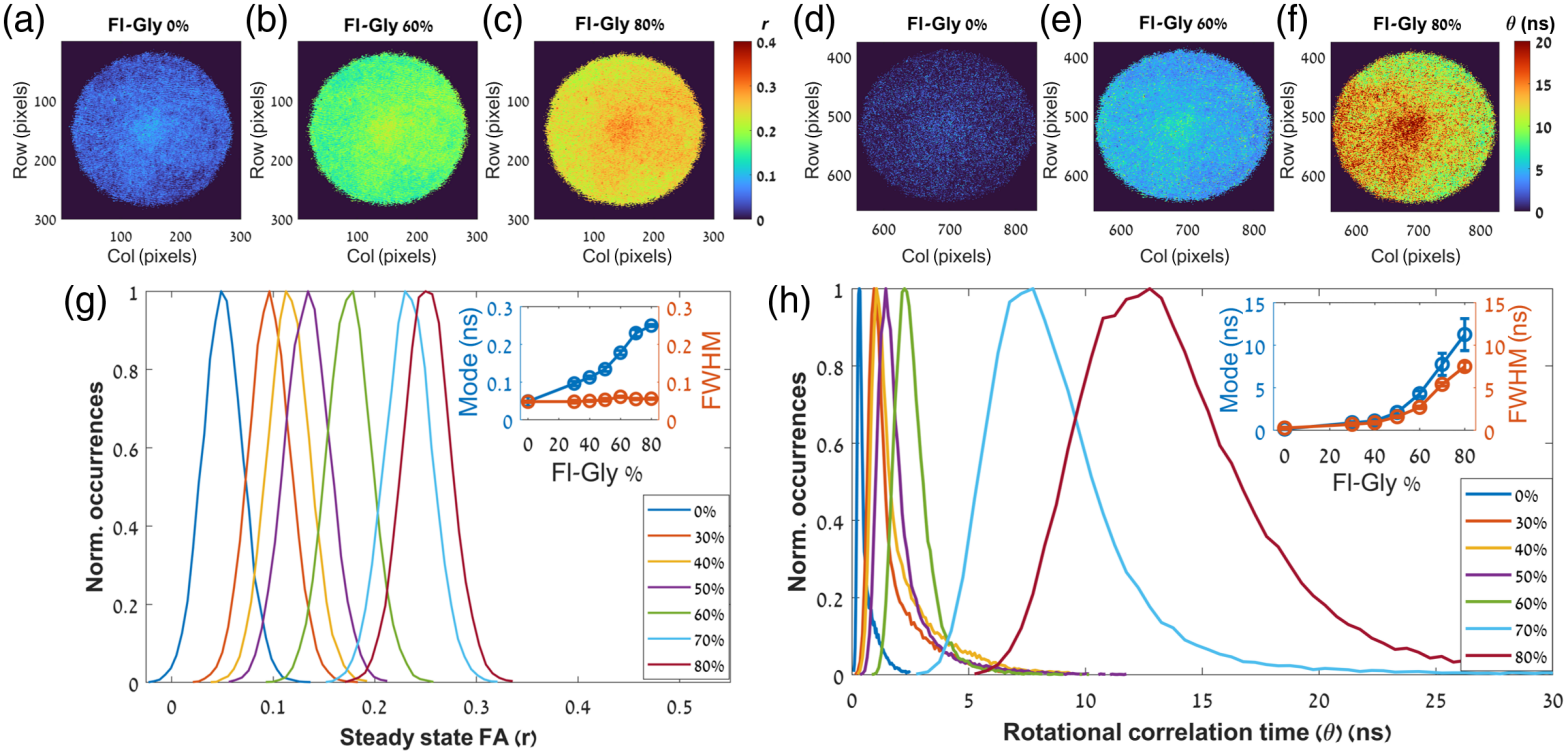
(a)–(c) Steady-state FA and (d-f) rotational correlation time maps of Fl-Gly solutions with different glycerol concentrations (0%, 60%, and 80%). The steady-state FA and the rotational correlation time are represented by a color scale from 0 to 0.4 and 0 to 20 ns, respectively. The increasing of glycerol concentration expressed by the increase of the steady-state FA and the rotational correlation time. Both are also presented by histograms of the steady-state FA (g) and the rotational correlation time (h) of Fl-Gly (with different glycerol concentrations between 0% and 80%). Insets: the modes of the histograms of both parameters (blue circles) show a clear increase with the increasing of glycerol concentration. However, whereas the FWHM of the steady-state FA distributions remains constant the FWHM of the rotational correlation time distributions also exhibit a clear increase (orange circles).

Next, the steady-state FA and the dynamic FA were also measured (the findings are shown in Fig. 3 and summarized in Table 1). While the increase of the glycerol concentration from the 0% to 80% resulted in a moderate decrease in the FLT (about 0.5 ns), it presented a strong dependence in both the steady state FA [Figs. 3(a)–3(c)] and the rotational correlation time [Figs. 3(d)–3(f)]. The former and the latter are expressed by the two orders of magnitude increase in the r values from 0.053 to 0.252 and the θ values from 0.15 ns to 11.25 ns [blue circles in the inset of Figs. 3(g) and 3(h), respectively]. These trends demonstrate the utility of imaging the rotational mobility beside the FLT as the former presented clearer distinctions between the samples. Moreover, although the increase in the viscosity was not followed by a wider distribution of r, it resulted in a wider distribution of θ [orange circles in the inset of Figs. 3(g) and 3(h), respectively] as evident by the increase of the FWHM from 0.27 ns for the 0% to 7.50 ns for 80%.

With the exception of the 0% solution, the other solutions (with 30% to 80%) exhibited higher correlation times and hence more viscous regions in the center of each image [as presented for example in Figs. 3(e) and 3(f)]. Since the center of each sample has been adjusted to the center of the illumination, this can be explained by the favorable peripheral diffusion of the less viscous liquid (fluorescein distilled in phosphate buffer saline) relative to the more viscous liquid (glycerol). This can justify why FLT maps did not reveal this phenomenon (the fluorescein was still relatively uniformly distributed) as well as why the pure fluorescein (without glycerol) did not exhibit this behavior. Moreover, it should be noted that the effective dynamic range of θs measured by FA decay is typically limited between 0.1 and 10 times the FLT without constrained analysis or a high level of signal to noise ratio.^62^ The mean θ for the 0% solution was about 0.40 ns, whereas the more concentrated solutions (30% to 80%) samples exhibited mean values between 1.5 and 12.5 ns. Therefore, the determination of θ for the 0% solution (with mean FLT about 4 ns) might be less accurate.

Moreover, the increase in the glycerol concentration resulted in a noticeable decrease in the fundamental FA, $r_{0}$ (Table 1). This implies an increase in the angle between the absorption and emission dipole moments of the fluorescein, due to changes of its molecular conformation with the glycerol concentration. All the investigated parameters (FLT, r, θ, and $r_{0}$) of the Fl-Gly samples are in good agreement with previously published cuvettes and single point measurements.^14^

### 3.2 Imaging CDs

Compared to other carbon nanostructures, CDs are fluorescent, photostable, and their optical properties are unique and tunable. Several mechanisms responsible for the change of fluorescence properties in CDs have already been applied for sensing applications.^63^^–^^65^ The near-field interactions of CDs and the AuNPs can cause the spectral emission of the fluorescence to change wavelengths making CDs-Au constructs suitable FLIM probes (as is evident by the fundamental Strickler-Berg formula,^2^ which is discussed in Appendix B in the Supplementary Material).

Although there are several works that characterize CDs using FI and FLT analyses, there are only a few reports that use steady-state FA analysis and to the best of our knowledge none that use θ imaging. However, the increasing size of the linked CDs-Au constructs relative to the CDs alone make them favorable for steady-state FA and θ imaging experiments as the increase in the effective size of the combined construct should increase the polarization [as evident in Eqs. (1) and (4)].

#### 3.2.1 Morphology and absorption spectrum of the CDs and CDs-Au

**Fig. 4:**
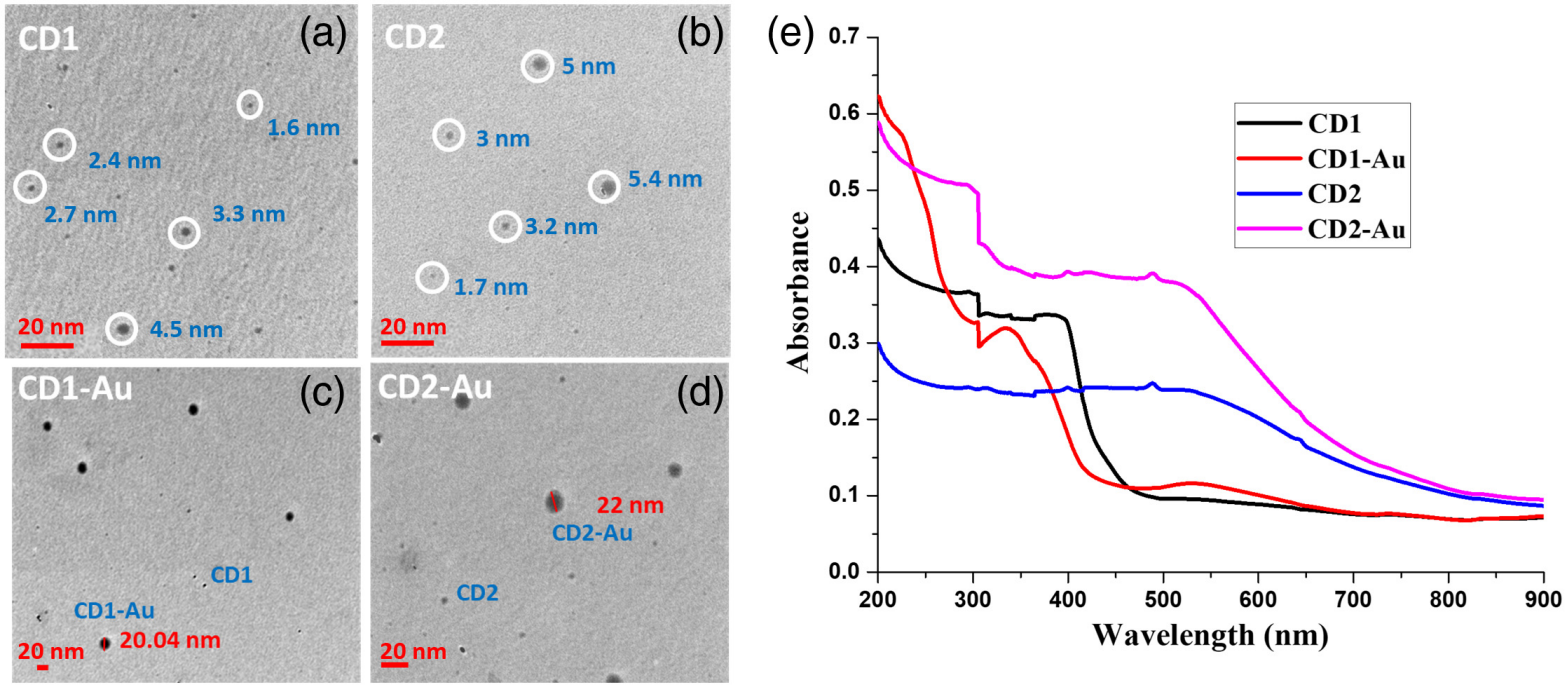
TEM images of (a) CD1, (b) CD2, (c) CD1-Au, and (d) CD2-Au nanohybrids. The 20 scale (bottom left in each subplot) has changed size in direct proportion to the physical size of the map. Clearly, there is an increase in the effective size of each CDs-Au construct relative to the CDs alone. (e) UV−VIS absorption spectra of CD1, CD1-Au, CD2, and CD2-Au.

To confirm the attachment of each of the CDs (as described in Sec. 2.5.1) to the AuNPs by the increase in the effective size of the CDs-Au construct relative to the CDs alone, the morphologies and dimensions of the CDs and CDs-Au nanohybrid were investigated by transmission electron microscopy (TEM, JEM-1400Flash, Massachusetts, United States). All CDs were determined to be spherical in shape, which explains why the single exponential decay model was found to best describe the FA decay. Nevertheless, the attachment to gold distinctly increased the sizes of CD1 and CD2 from 2 to 5 nm to 20 to 22 nm for the CD1-Au and CD2-Au constructions, respectively [Figs. 4(a)–4(d)]. The TEM images for the AuNPs alone found a size range from 14 to 18 nm [Fig. S2(a) in Appendix D in the Supplementary Material]. In addition, a prominent peak in the CDs Fourier transform infra-red spectroscopy (FTIR, Perkin Elmer, Spectrum Two, Singapore) spectra [Fig. S2(b) and Appendix D in the Supplementary Material] at $1515\;{\mathrm{cm}}^{-1}$ indicated the existence of the -NH group, whereas a broad peak at $1345\;{\mathrm{cm}}^{-1}$ indicated OH deformation vibrations. The change of the carbonyl peak of carboxylic acid from 1623 to $1609\;{\mathrm{cm}}^{-1}$ endorsing the establishment of an amide linkage confirmed the covalent attachment of AuNPs to the CD surface.^66^ The verification of both the increased size of the CDs-Au nanohybrid relative to the CDs alone and the covalent attachment of AuNPs to the CD surface imply that rotational correlation time imaging should distinguish between each of the two CDs and their attachment to gold.

The CDs UV–visible (VIS) spectra showed a wide spectrum throughout the whole region, with the largest peaks occurring at 337 and 517 nm [Fig. 4(e)]. The graphitic sp2 domain of CDs underwent a Π-Π* transition,^67^ which led to the observation of the absorption band at 337 nm. The aromatic core made up of C-O or C-N structures may have undergone n-Π* transitions, which would explain the peak at 517 nm.^62^^,^^68^

Since all CDs were excited by 468 nm, observation of the absorption spectrum of the CDs can imply several theses. First, the FI is expected to be higher for CD2 compared to CD1 since the absorbance is more than two times stronger for the 468 nm. Second, if CD2 had undergone n-Π* transitions, it should be reflected by larger $r_{0}$ values relative to CD1 and even the maximal $r_{0}$ value ∼0.4 (absorption and emission involving the same electronic transition have nearly colinear moments whereas lower $r_{0}$ values are expected upon excitation into higher electronic states).^69^ Third, for the same reason, CD2 may have higher steady state FA (r) relative to CD1.

#### 3.2.2 Fluorescence imaging of the CDs and CDs-Au

This section tests the above hypotheses and presents the fluorescence properties (FI, FLT, r, θ, and $r_{0}$) of the two CDs derived from different carbon source (as elaborated in Sec. 2.5.1). Moreover, each of the two CDs was investigated with and without attachment to gold (Secs. 2.5.2 and 2.5.3).

**Fig. 5:**
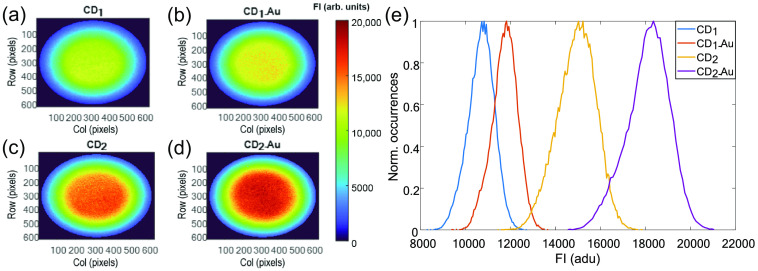
(a)–(d) FI maps and (e) histograms of the four CDs (the FI is represented by a color scale from 0 to 20000 arb. units). CD1 and CD2 differ only by the carbon source (CA in CD1 and p-PD in CD2). Distinctly, CD2 presented higher FI than CD1 and the attachment of gold to each CD increase the FI.

Expectedly (first thesis in Sec. 3.2.1), the FI images [Figs. 5(a)–5(d)] and histograms [Fig. 5(e)] of the CDs indicate higher values for CD2 relative to CD1. However, surprisingly, each of the CDs exhibited higher FI through the attachment to gold. In addition, as presented in Fig. 5(e) and summarized in Table 2, the FI histograms reveal wider distribution (higher FWHM) for CD2 relative to CD1. Nonetheless, the attachment of gold did not alter the FWHM.

**Table 2:** Characterization of FI and FI decays of the four CDs (0% to 80%).

	FI (arb. units)		FLT (ns)^*^	
Sample	Mode	FWHM	Mode	FWHM
CD1	10,825 ± 620	1125 ± 570	2.66 ± 0.10	0.36 ± 0.02
CD1-Au	11,825 ± 570	1100 ± 850	2.62 ± 0.05	0.33 ± 0.02
CD2	15,225 ± 625	2000 ± 400	2.47 ± 0.19	0.20 ± 0.05
CD2-Au	18,350 ± 1070	2000 ± 400	2.42 ± 0.06	0.21 ± 0.04

* Since typically the FLT has standard deviation < 0.2, it is exhibited with an accuracy of 2 decimal place.^47^

**Fig. 6:**
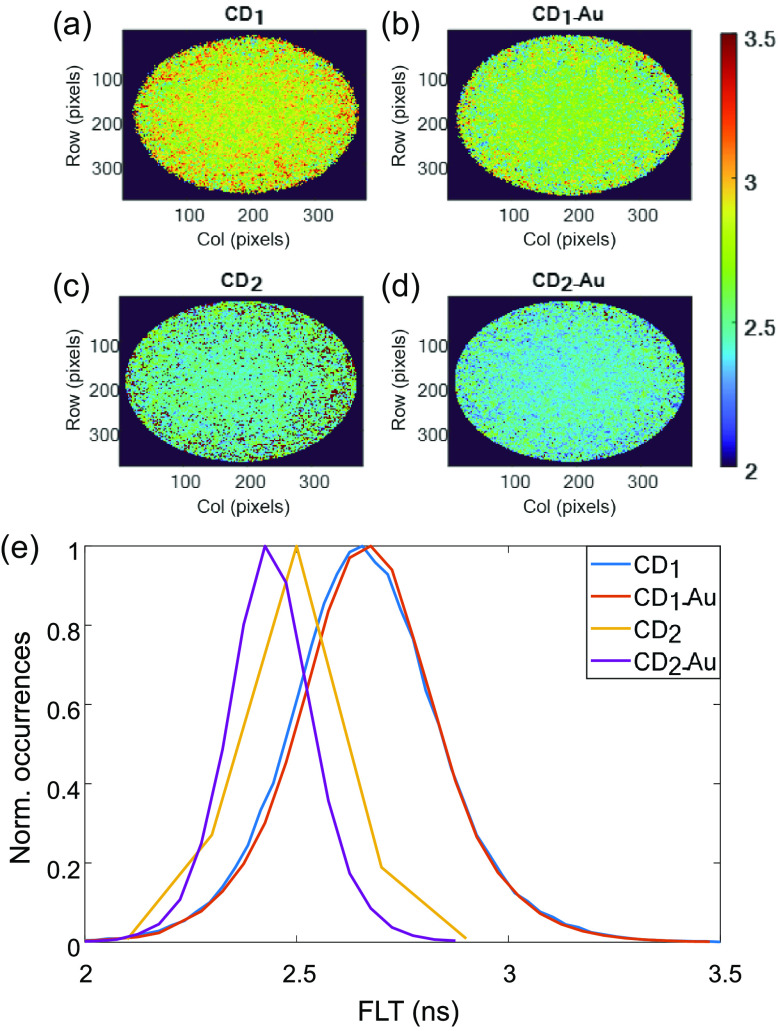
(a)–(d) FLT maps and (e) histograms of the CDs (the FLT is represented by a color scale from 2 to 3.5 ns). Clearly, CD1 exhibited longer FLT relate to CD2. However, the attachment of gold to CD1 and CD2 barely decreased the FLT (within experimental errors).

Next, FLIM was applied on the CDs [presented in Figs. 6(a)–6(d) and summarized in Table 2], an observable shift to shorter FLT values in CD2 relative to CD1 and in the addition of gold to CD2 was seen. However, there is a barely distinguishable shift with the addition of gold in CD1. FLIM histograms [Fig. 6(e)] also reveal narrower FLT distribution (lower FWHM) in CD2 relative to CD1 but about the same distributions with and without the attachment to gold (Table 2). Moreover, as opposed to the homogeneous environments of the Fl-Gly samples, which are reflected by relatively uniform FLT images, the varying environments across the CDs exhibited more detailed FLT images. Although the FI histograms visibly distinguish between the four CDs, the FI images did not reveal environmental sensitivity. This demonstrates how simultaneously imaging the FI and the FLT can provide complementary information.

**Fig. 7:**
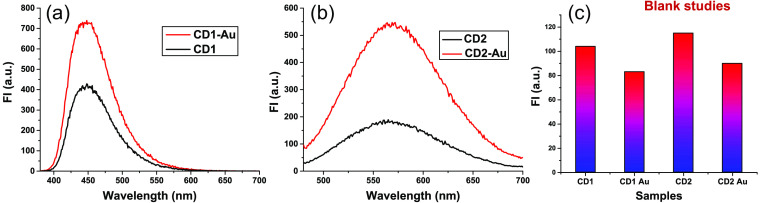
(a) Emission spectra of CD1 and (b) CD2, respectively, in the presence of AuNPs showing MEF. (c) Blank studies without PEI coating on CD1 and CD2 showing no MEF.

The modest FLT variation due to the attachment to gold of each CD can indicate that the increase in FI [Fig. 5(e)] with the attachment to gold is not caused by the inhibition of quenching (which should appreciably decrease the FLT) but by metal-enhanced fluorescence (MEF). To support this hypothesis, the fluorescence emission spectra of CD1, CD2, with and without the presence of AuNPs, were captured and compared to confirm the MEF [Figs. 7(a) and 7(b)]. The presence of amine PEG coated AuNPs [Fig. S2(a) and Appendix D in the Supplementary Material] was discovered to improve the FI. When AuNPs were attached to CDs without PEI coating (blank studies), we noticed a slight decrease in FI [Fig. 7(c)]. Thus, we concluded that the MEF effect, which is enforced by AuNPs in a proper configuration with PEI coating on CDs, is certainly responsible for the enhancement.

Finally, we utilized a TD system equipped with a scanning confocal PicoQuant micro time microscope (MT200, PicoQuant, Berlin, Germany) and the ability to perform time-correlated single-photon counting to measure the FLT. The findings indicate that CD1 had an FLT of 2.8±0.042, CD1-Au had an FLT of 2.6±0.090  ns, CD2 had an FLT of 2.4±0.095, and CD2-Au had an FLT of 2.45±0.045. These FLT measurements are comparable to those obtained using the FD FLIM system.

**Fig. 8:**
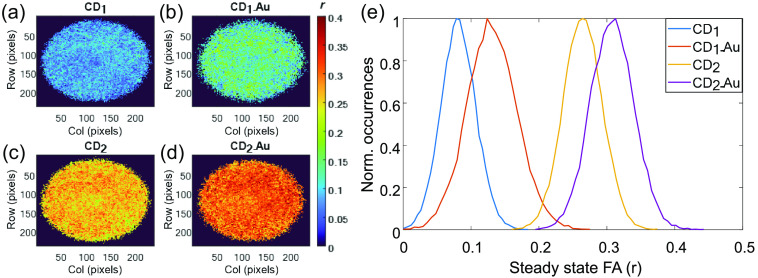
(a)–(d) Steady state FA maps and (e) histograms of the CDs (the r is represented by a color scale from 0 to 0.4). Unquestionably, CD2 exhibited higher FA relative to CD1 as well as each CD presented higher FA due to the attachment to gold.

Next, the FD TR-FAIM configuration was used to study both the CDs in terms of steady state and dynamic FA, both can further verify whether CD2 undergone a n-Π* transition or a Π-Π* transition like CD1 as well as the size alteration of each CD with the link to gold. The steady state FA maps, as presented in Figs. 8(a)–8(d), and histograms [Fig. 8(e)] reveal higher polarization in CD2 relative to CD1 and higher polarization in each CD with the attachment to gold. Both the former and the latter imply that simple steady state FA analysis is sufficient to distinguish between the two carbon sources and verify the coupling of CDs to AuNPs (using the EDC-NHS), respectively. Moreover, CD2 also presented a broader distribution compared to CD1 and the addition of gold to each CD decreased the FWHM (Table 3).

**Table 3:** Characterization of steady-state FA and FA decays of the four CDs.

	r *		θ (ns)		$r_{0}$
Sample	Mode	FWHM	Mode	FWHM	Mode
CD1	0.100 ± 0.011	0.075 ± 0.011	0.98 ± 0.13	0.66 ± 0.01	0.16 ± 0.01
CD1-Au	0.150 ± 0.013	0.065 ± 0.005	1.65 ± 0.20	1.10 ± 0.13	0.23 ± 0.05
CD2	0.280 ± 0.008	0.078 ± 0.009	5.55 ± 1.08	4.26 ± 0.47	0.36 ± 0.01
CD2-Au	0.310 ± 0.004	0.055 ± 0.008	7.95 ± 0.97	5.30 ± 0.68	0.40 ± 0.01

* Since typically r has standard deviation <0.01, it is exhibited with an accuracy of 3 decimal place, whereas θ and $r_{0}$ are exhibited with an accuracy of 2 decimal place as the FLT.

**Fig. 9:**
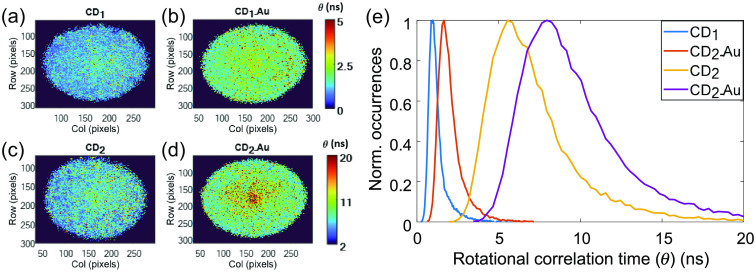
(a)–(d) Rotational correlation time maps and (e) histograms of the CDs (θ is represented by a color scale from 0 to 5 ns for CD1 and 2 to 20 ns for CD2). In accordance with the findings of the steady state FA, CD2 exhibited higher θ relative to CD1 and the increase of θ in each CD due to the attachment to gold is evident.

Expectedly, the general trends seen through steady state FA imaging were repeated using correlation time imaging [Figs. 9(a)–9(d) and Table 3] and observing its histograms [Fig. 9(e)]. CD2 exhibited higher correlation times relative to CD1 and the attachment to gold also increased the correlation time in each CD. However, two main additional findings demonstrated the superiority of correlation time imaging. First, as evident in Table 3, the FWHM followed the same trends as the mode (contrary to the steady state FA investigations). Second, following the difference between the FI imaging (Fig. 3) and the FLT imaging (Fig. 5), the correlation time imaging provided more detailed images relative to the steady state FA images (Fig. 8). In agreement with the Fl-Gly maps [Figs. 3(e) and 3(f)], the correlation time images of the CDs revealed a more viscous area in the center of each image. This supports the thesis that in each sample the less viscous fluid tends to diffuse more rapidly to the peripheral area (all CDs were dissolved in water, which diffused faster).

Finally, the fluorescence characteristics of the steady-state FA and FA decay are summarized in Table 3 using the mode and FWHM of each parameter distribution. Moreover, the mode of the fundamental FA ($r_{0}$) is also presented. CD2 exhibited about collinear absorption and emission transmission moments (indicated by about maximal value of 0.4), contrary to CD1, which exhibited lower fundamental FA. This supports the second thesis (end of Sec. 3.2.1) that the absorption and emission in CD2 involving the same electronic transition and hence have nearly colinear moments, whereas the excitation in CD1 is into higher electronic state (n-Π* transition in CD2 vs Π -Π* transmission in CD1).

## 4 Conclusion and Future Work

The present work demonstrates how the FD TR-FAIM system can serve as a powerful tool for performing parallel imaging based on rotational diffusion dynamics and excited states kinetics. Conceivably, a large variety of unique information can be extracted with great sensitivity on a pixel by- pixel basis by combining the steady-state images (FI and r) and the TR images (FLT and θ) obtained by FD FLIM/ TR-FAIM at multiple modulation frequencies. Measuring the FLT and θ in the FD has the potential to shed light on distinct photophysical characteristics or microenvironments in a rapid and efficient manner.

In the present research, the main fluorescence characteristics (FI, r, FLT, and θ) were investigated using the combined FD FLIM/ TR-FAIM in seven Fl-Gly samples with increasing glycerol concentration and hence with increasing the viscosity. Investigation of each of the fluorescence characteristic had its own contribution toward describing the change in the fluorophore. Nevertheless, with a focus on the rotational correlation time imaging and investigation, a large variety of favorable and beneficial information can be obtained. Next, the FD FLIM/ TR-FAIM was used for comprehensive study of two different CDs distinguished by their carbon source, and each was studied with and without attachment to gold. Again, although generally each fluorescence characteristic was sensitive to the difference between the CDs, the change in the rotational correlation time was the most dominant.

In the FD TR-FAIM, the images of both polarization components were acquired simultaneously and hence less susceptible to inaccuracies due to instrumental and environmental factors. Consequently, the limiting speed factor of our system is currently the data processing of the acquired images. The main limitation of this system is that for proper imaging of the FA decay, one must monitor and assess each instrumental or environmental factor that can lead to polarization distortion. These include suboptimal modulation frequencies, high numerical aperture, noise level, scattering, photobleaching, or simply biases or birefringence in the optical components (filters, polarizers, reflectors, detectors, and more).^17^^,^^27^

Unfortunately, although two decades have passed since the revolutionary work of Clayton et al. in the FD (2002)^27^ and J. Siegel et al. in the TD (2003),^4^ correlation time imaging is still in its infancy. Nevertheless, FD TR-FAIM has a potential promise for many biological applications such as the studies of local microviscosity, complex formation, and molecular proximity in the molecular and cellular levels. Thus, correlation time imaging is likely to become more widely used, especially with the advance in technology.

While previous studies have shown the potential of TR-FAIM for cellular imaging, our research specifically targets CDs and AuNPs, which have not been studied in this context before to our knowledge. These materials offer unique features that are relevant to a wide range of research fields. Although our focus is on CDs and AuNPs, our findings demonstrate principles that can be applied to other cellular imaging applications using TR-FAIM.

## Code, Data, and Materials Availability

Code, data, and materials associated with this article are available upon request sent to the corresponding author.

## References

[1] Datta R. et al., “Fluorescence lifetime imaging microscopy: fundamentals and advances in instrumentation, analysis, and applications,” J. Biomed. Opt. 25(7), 071203 (2020). JBOPFO 1083-3668 10.1117/1.JBO.25.7.071203 32406215 PMC7219965

[2] Berezin M. Y. Achilefu S., “Fluorescence lifetime measurements and biological imaging,” Chem. Rev. 110(5), 2641–2684 (2010). CHREAY 0009-2665 10.1021/cr900343z 20356094 PMC2924670

[3] Suhling K. et al., “Fluorescence lifetime imaging (FLIM): basic concepts and some recent developments,” Med. Photonics 27, 3–40 (2015). 10.1016/j.medpho.2014.12.001

[4] Siegel J. et al., “Wide-field time-resolved fluorescence anisotropy imaging (TR-FAIM): imaging the rotational mobility of a fluorophore,” Rev. Sci. Instrum. 74(1), 182–192 (2003). RSINAK 0034-6748 10.1063/1.1519934

[5] Zhang H. et al., “Dual-emissive phosphorescent polymer probe for accurate temperature sensing in living cells and zebrafish using ratiometric and phosphorescence lifetime imaging microscopy,” ACS Appl. Mater. Interfaces 10(21), 17542–17550 (2018). AAMICK 1944-8244 10.1021/acsami.8b01565 29733202

[6] Lin H. J. Herman P. Lakowicz J. R., “Fluorescence lifetime-resolved pH imaging of living cells,” Cytometry Part A 52(2), 77–89 (2003).1552-4922 10.1002/cyto.a.10028 PMC6906609 12655651

[7] Tregidgo C. L. Levitt J. A. Suhling K., “Effect of refractive index on the fluorescence lifetime of green fluorescent protein,” J. Biomed. Opt. 13(3), 031218 (2008). JBOPFO 1083-3668 10.1117/1.2937212 18601542

[8] Saxl T. et al., “A fluorescence lifetime-based fibre-optic glucose sensor using glucose/galactose-binding protein,” Analyst 136(5), 968–972 (2011). ANLYAG 0365-4885 10.1039/C0AN00430H 21165474

[9] Penjweini R. et al., “Intracellular oxygen mapping using a myoglobin-mCherry probe with fluorescence lifetime imaging,” J. Biomed. Opt. 23(10), 107001 (2018). JBOPFO 1083-3668 10.1117/1.JBO.23.10.107001 30298706 PMC6210794

[10] Yahav G. et al., “Fluorescence lifetime imaging of DAPI-stained nuclei as a novel diagnostic tool for the detection and classification of B-cell chronic lymphocytic leukemia,” Cytometry Part A 89(7), 644–652 (2016).1552-4922 10.1002/cyto.a.22890 27315046

[11] Zahavi T. et al., “Utilizing fluorescent life time imaging microscopy technology for identify carriers of BRCA2 mutation,” Biochem. Biophys. Res. Commun. 480(1), 36–41 (2016). BBRCA9 0006-291X 10.1016/j.bbrc.2016.10.013 27721065

[12] Gershanov S. et al., “Fluorescence lifetime imaging microscopy, a novel diagnostic tool for metastatic cell detection in the cerebrospinal fluid of children with medulloblastoma,” Sci. Rep. 7, 3648 (2017). SRCEC3 2045-2322 10.1038/s41598-017-03892-6 28623325 PMC5473849

[13] Chakraborty S. et al., “Quantification of the metabolic state in cell-model of Parkinson’s disease by fluorescence lifetime imaging microscopy,” Sci. Rep. 6, 19145 (2016). SRCEC3 2045-2322 10.1038/srep19145 26758390 PMC4725947

[14] Fixler D. et al., “Tracing apoptosis and stimulation in individual cells by fluorescence intensity and anisotropy decay,” J. Biomed. Opt. 10(3), 034007 (2005). JBOPFO 1083-3668 10.1117/1.1924712 16229651

[15] Fixler D. Nayhoz T. Ray K., “Diffusion reflection and fluorescence lifetime imaging microscopy study of fluorophore-conjugated gold nanoparticles or nanorods in solid phantoms,” ACS Photonics 1(9), 900–905 (2014). 10.1021/ph500214m 25541621 PMC4270410

[16] Lakowicz J. R., “Time-dependent anisotropy decays,” in Principles of Fluorescence Spectroscopy, Lakowicz J., ed., pp. 383–412, Springer Science+Business Media LLC, New York, NY, USA (2013).

[17] Vogel S. S. et al., “Time-resloved fluorescence anisotropy,” in FLIM Microscopy in Biology and Medicine, Periasamy A. Clegg R. M., Eds., pp. 277–320, Chapman and Hall/CRC (2009).

[18] Hutchinson R. et al., “A novel approach combining fluorescence-anisotropy decays and microviscometry to explore the cotranslational compaction of nascent proteins,” Biophys. J. 120(3), 99a (2021). BIOJAU 0006-3495 10.1016/j.bpj.2020.11.816

[r19] Lakowicz J. R., “Fluorescence spectroscopic investigations of the dynamic properties of proteins, membranes and nucleic acids,” J. Biochem. Biophys. Methods 2(1-2), 91–119 (1980). JBBMDG 0165-022X 10.1016/0165-022X(80)90077-9 6158533

[20] Ojha N. Rainey K. H. Patterson G. H., “Imaging of fluorescence anisotropy during photoswitching provides a simple readout for protein self-association,” Nat. Commun. 11, 21 (2020). NCAOBW 2041-1723 10.1038/s41467-019-13843-6 31911590 PMC6946710

[21] Bhuyan N. N. et al., “Exploring membrane viscosity at the headgroup region utilizing a hemicyanine-based fluorescent probe,” J. Mol. Liquids 325, 115–152 (2021). JMLIDT 0167-7322 10.1016/j.molliq.2020.115152

[22] Edmonds D. M., “Fluorescence polarization immunoassay diagnostic method,” Google Patents (2000).

[23] Vinegoni C. et al., “Fluorescence anisotropy imaging in drug discovery,” Adv. Drug Deliv. Rev. 151, 262–288 (2019). ADDREP 0169-409X 10.1016/j.addr.2018.01.019 29410158 PMC6072632

[24] Silori Y. De A. K., “Controlling balance between homo-FRET and hetero-FRET within hetero-chromophoric systems by tuning nature of solvent,” J. Mol. Liquids 298, 112093 (2020). JMLIDT 0167-7322 10.1016/j.molliq.2019.112093

[25] Teijeiro-Gonzalez Y. et al., “Time-resolved fluorescence anisotropy and molecular dynamics analysis of a novel GFP homo-FRET dimer,” Biophys. J. 120(2), 254–269 (2021). BIOJAU 0006-3495 10.1016/j.bpj.2020.11.2275 33345902 PMC7840444

[26] Suhling K. et al., “Time-resolved fluorescence anisotropy imaging applied to live cells,” Opt. Lett. 29(6), 584–586 (2004). OPLEDP 0146-9592 10.1364/OL.29.000584 15035478

[r27] Clayton A. H. et al., “Dynamic fluorescence anisotropy imaging microscopy inthe frequency domain (rFLIM),” Biophys. J. 83(3), 1631–1649 (2002). BIOJAU 0006-3495 10.1016/S0006-3495(02)73932-5 12202387 PMC1302260

[r28] Zheng K. et al., “Nanoscale diffusion in the synaptic cleft and beyond measured with time-resolved fluorescence anisotropy imaging,” Sci. Rep. 7(1), 1–10 (2017). SRCEC3 2045-2322 10.1038/srep42022 28181535 PMC5299514

[r29] Levitt J. A. et al., “Simultaneous FRAP, FLIM and FAIM for measurements of protein mobility and interaction in living cells,” Biomed. Opt. Express 6(10), 3842–3854 (2015). BOEICL 2156-7085 10.1364/BOE.6.003842 26504635 PMC4605044

[r30] Teijeiro-Gonzalez Y. et al., “Fluorescence recovery after photobleaching (FRAP) with simultaneous fluorescence lifetime and time-resolved fluorescence anisotropy imaging (FLIM and tr-FAIM),” Proc. SPIE 10883, 108830A (2019). PSISDG 0277-786X 10.1117/12.2508692

[31] Zhou Y. Dickenson J. Hanley Q., “Imaging lifetime and anisotropy spectra in the frequency domain,” J. Microsc. 234(1), 80–88 (2009). JMICAR 0022-2720 10.1111/j.1365-2818.2009.03145.x 19335458

[32] Ross J. A. Jameson D. M., “Time-resolved methods in biophysics. 8. Frequency domain fluorometry: applications to intrinsic protein fluorescence,” Photochem. Photobiol. Sci. 7(11), 1301–1312 (2008). PPSHCB 1474-905X 10.1039/b804450n 18958316

[r33] Lakowicz J. R., “Frequency-domain lifetime measurements,” in Principles of Fluorescence Spectroscopy, Lakowicz J., ed., pp. 158–203, Springer Science+Business Media LLC, New York, USA (2013).

[r34] Chen X. et al., “Dual-modality optical coherence tomography and frequency-domain fluorescence lifetime imaging microscope system for intravascular imaging,” J. Biomed. Opt. 25(9), 096010 (2020). JBOPFO 1083-3668 10.1117/1.JBO.25.9.096010 33000570 PMC7525154

[r35] Sumetsky D. et al., “Linear behavior of the phase lifetime in frequency-domain fluorescence lifetime imaging of FRET constructs,” Mod. Tools Time-Resolved Lumin. Biosens. Imaging 9, 648016 (2021). 10.3389/fphy.2021.648016

[r36] Hutchinson R. B. et al., “Fluorescence anisotropy decays and microscale-volume viscometry reveal the compaction of ribosome-bound nascent proteins,” J. Phys. Chem. B 125(24), 6543–6558 (2021). JPCBFK 1520-6106 10.1021/acs.jpcb.1c04473 34110829 PMC8741338

[37] Reichert D. et al., “Improved protoporphyrin IX-guided neurosurgical tumor detection with frequency-domain fluorescence lifetime imaging,” Appl. Sci. 12(3), 1002 (2022). 10.3390/app12031002

[38] Lakowicz J. R. et al., “Fluorescence lifetime imaging,” Anal. Biochem. 202(2), 316–330 (1992). ANBCA2 0003-2697 10.1016/0003-2697(92)90112-K 1519759 PMC6986422

[39] Chan F. T. Kaminski C. F. Kaminski Schierle G. S., “HomoFRET fluorescence anisotropy imaging as a tool to study molecular self-assembly in live cells,” ChemPhysChem 12(3), 500–509 (2011). CPCHFT 1439-4235 10.1002/cphc.201000833 21344590

[40] Pu Y. et al., “Time-resolved fluorescence polarization anisotropy and optical imaging of Cybesin in cancerous and normal prostate tissues,” Opt. Commun. 274(1), 260–267 (2007). OPCOB8 0030-4018 10.1016/j.optcom.2007.01.078

[41] Hirvonen L. M. et al., “Hydrodynamic radii of ranibizumab, aflibercept and bevacizumab measured by time-resolved phosphorescence anisotropy,” Pharm. Res. 33(8), 2025–2032 (2016). PHREEB 0724-8741 10.1007/s11095-016-1940-2 27225494 PMC4942501

[42] Wang X. et al., “A mini review on carbon quantum dots: preparation, properties, and electrocatalytic application,” Front. Chem. 7, 671 (2019). FCBIE2 10.3389/fchem.2019.00671 31637234 PMC6787169

[43] Pawar S. et al., “Carbon dots-based logic gates,” Nanomaterials 11(1), 232 (2021). 10.3390/nano11010232 33477327 PMC7830989

[r44] Wang B. et al., “Carbon dots in bioimaging, biosensing and therapeutics: a comprehensive review,” Small Sci. 2(6), 2200012 (2022). 10.1002/smsc.202200012 PMC11935858 40213786

[45] Long C. et al., “Applications of carbon dots in environmental pollution control: a review,” Chem. Eng. J. 406, 126848 (2021). 10.1016/j.cej.2020.126848

[46] Barnoy E. A. et al., “Biological logic gate using gold nanoparticles and fluorescence lifetime imaging microscopy,” ACS Appl. Nano Mater. 2(10), 6527–6536 (2019). 10.1021/acsanm.9b01457

[47] Van Geest L. Stoop K., “FLIM on a wide field fluorescence microscope,” Lett. Peptide Sci. 10(5–6), 501–510 (2003). 10.1007/BF02442582

[48] Jablonski A., “On the notion of emission anisotropy,” Bull. Acad. Pol. Sci. 8, 259–264 (1960). BAPTA9 0001-4125

[49] Perrin F., “Polarisation de la lumière de fluorescence. Vie moyenne des molécules dans l’etat excité,” J. Phys. Radium 7(12), 390–401 (1926). JPRAAJ 0368-3842 10.1051/jphysrad:01926007012039000

[50] Lakowicz J. R. et al., “Review of fluorescence anisotropy decay analysis by frequency-domain fluorescence spectroscopy,” J. Fluoresc. 3(2), 103–116 (1993). JOFLEN 1053-0509 10.1007/BF00865324 24234774

[51] Fixler D. et al., “Influence of fluorescence anisotropy on fluorescence intensity and lifetime measurement: theory, simulations and experiments,” IEEE Trans. Biomed. Eng. 53(6), 1141–1152 (2006). IEBEAX 0018-9294 10.1109/TBME.2006.873539 16761841

[52] Atabaev T. S., “Doped carbon dots for sensing and bioimaging applications: a mini review,” Nanomaterials 8(5), 342 (2018). 10.3390/nano8050342 29783639 PMC5977356

[53] Han B. et al., “Polyethyleneimine modified fluorescent carbon dots and their application in cell labeling,” Colloids Surf. B Biointerfaces 100, 209–214 (2012). 10.1016/j.colsurfb.2012.05.016 22766299

[54] Liu C. et al., “Nano-carrier for gene delivery and bioimaging based on carbon dots with PEI-passivation enhanced fluorescence,” Biomaterials 33(13), 3604–3613 (2012). BIMADU 0142-9612 10.1016/j.biomaterials.2012.01.052 22341214

[55] Taylor J. Adams M. Sibbett W., “Investigation of viscosity dependent fluorescence lifetime using a synchronously operated picosecond streak camera,” Appl. Phys. 21(1), 13–17 (1980). APPYEK 1080-9198 10.1007/BF00886477

[56] Kuimova M. K. et al., “Molecular rotor measures viscosity of live cells via fluorescence lifetime imaging,” J. Am. Chem. Soc. 130(21), 6672–6673 (2008). JACSAT 0002-7863 10.1021/ja800570d 18457396

[57] Haidekker M. A. et al., “Sensing of flow and shear stress using fluorescent molecular rotors,” Sens. Lett. 3(1-2), 42–48 (2005). 10.1166/sl.2005.003

[58] Strickler S. Berg R. A., “Relationship between absorption intensity and fluorescence lifetime of molecules,” J. Chem. Phys. 37(4), 814–822 (1962). JCPSA6 0021-9606 10.1063/1.1733166

[59] Toptygin D. et al., “Effect of the solvent refractive index on the excited-state lifetime of a single tryptophan residue in a protein,” J. Phys. Chem. B 106(14), 3724–3734 (2002). JPCBFK 1520-6106 10.1021/jp0133889

[60] Bünau G. V., JB Birks: Photophysics of Aromatic Molecules, Wiley-Interscience, Wiley Online Library (1970).

[61] Magde D. Rojas G. E. Seybold P. G., “Solvent dependence of the fluorescence lifetimes of xanthene dyes,” Photochem. Photobiol. 70(5), 737–744 (1999). PHCBAP 0031-8655 10.1111/j.1751-1097.1999.tb08277.x

[62] Sun S. et al., “Toward high-efficient red emissive carbon dots: facile preparation, unique properties, and applications as multifunctional theranostic agents,” Chem. Mater. 28(23), 8659–8668 (2016). CMATEX 0897-4756 10.1021/acs.chemmater.6b03695

[63] Guo Y. et al., “Fluorescent carbon nanoparticles for the fluorescent detection of metal ions,” Biosens. Bioelectron. 63, 61–71 (2015). BBIOE4 0956-5663 10.1016/j.bios.2014.07.018 25058940

[r64] Formica M. et al., “New fluorescent chemosensors for metal ions in solution,” Coord. Chem. Rev. 256(1-2), 170–192 (2012). CCHRAM 0010-8545 10.1016/j.ccr.2011.09.010

[65] Pawar S. et al., “Design and use of a gold nanoparticle–carbon dot hybrid for a FLIM-based implication nano logic gate,” ACS Omega 7(26), 22818–22824 (2022). 10.1021/acsomega.2c02463 35811911 PMC9260748

[66] Mitra T. et al., “Studies on cross-linking of succinic acid with chitosan/collagen,” Mater. Res. 16, 755–765 (2013). 10.1590/S1516-14392013005000059

[67] Cai W. et al., “Full color carbon dots through surface engineering for constructing white light-emitting diodes,” J. Mater. Chem. C 7(8), 2212–2218 (2019). 10.1039/C9TC00274J

[68] Zhang J. et al., “Scale-up synthesis of fragrant nitrogen-doped carbon dots from bee pollens for bioimaging and catalysis,” Adv. Sci. 2(4), 1500002 (2015). 10.1002/advs.201500002 PMC5115353 27980929

[69] Lakowicz J. R., “Fluorescence Anisotropy,” in Principles of Fluorescence Spectroscopy, Lakowicz J., ed., pp. 353–381, Springer Science+Business Media LLC, New York, NY, USA (2013).

