# Supplementary material for: Imaging the rotational mobility of carbon dot-gold nanoparticle conjugates using frequency domain wide-field time-resolved fluorescence anisotropy
Source: J Biomed Opt. 2023 May 23;28(5):056001. doi: 10.1117/1.JBO.28.5.056001 (PMC10203731; doi:10.1117/1.JBO.28.5.056001)
Supplement: Supplementary file 1 [file JBO_028_056001_SD001.pdf]

Imaging the rotational mobility of carbon dot-gold nano particle conjugates using frequency domain wide-field time-resolved fluorescence anisotropy

Gilad Yahav, Shweta Pawar, Yitzchak Weber, Bar Atuar, Hamootal Duadi, Dror Fixler*
The Faculty of Engineering and the Institute of Nanotechnology and Advanced Materials, Bar Ilan University, Ramat Gan, Israel.

Appendix A

This section describes the derivation of the equations for correcting the modulated intensity leakage in the PBS of the transmitted polarized light ($I_{\parallel}$) into the reflected polarized light ($I_{\perp}$).

In order to extract the steady state and dynamic FA images in our particular setup, the amount of leakage (L[%]) of the transmitted polarized component to the reflected component should be taken into account.

Prior to utilizing the polarized beam splitter, we conducted measurements on four fluorescein-glycerol solutions (containing 0%, 30%, 60%, and 80% glycerol) using a linear polarizer in the emission path oriented both parallel ($0^0/0^0$-excitation/emission) and perpendicular ($0^0/90^0$) to the excitation vertical orientation. Upon investigating the impact of replacing the linear polarizer with a polarizing beam splitter, we noted that the manufacturer indicated a possible leakage of up to 5%. After repeating the measurements with the polarizing beam splitter, we observed a clear decrease in the *r* values. We then derived formulas to compensate for the leakage and discovered that a 5% leakage resulted in good agreement between the results obtained using a linear polarizer in the emission path and those obtained using the polarizing beam splitter.

Considering in the PBS the 98% transmission of the reflected polarization (PBS and mirror) and 90% transmission of the transmitted polarization, the corrected intensity of the reflected polarization ($I_{\perp}^{corr}$) is given by

$$I_{\perp}^{corr} = 1.02 I_{\perp}^{\exp} - \frac{10}{9} L I_{\parallel}^{\exp}, \tag{S1}$$

where L is the amount of leakage in % (5 in our system). $I_{\perp}^{\exp}$ and $I_{\parallel}^{\exp}$ are the measured perpendicular and parallel intensities. The explicit terms of the modulated intensities are of:

$$I = I^{DC}[1 + m\sin(\omega t - \phi)] = I^{DC} + I^{AC}\sin(\omega t - \phi), \tag{S2}$$

where I^{DC} and I^{AC} are the DC and AC components of the fluorescence intensity, *m* is the modulation and ϕ is the phase. Substitution of Eq. (S2) in Eq. (S1) yields that the DC component can be extracted setting $I = I^{DC}$ in Eq. (S1), and the AC component by

$$I_{\perp}^{AC,corr}\sin(\omega t - \phi_{\perp}^{corr}) = 1.02 I_{\perp}^{DC,\exp} m_{\perp}^{\exp}\sin(\omega t - \phi_{\perp}^{\exp}) - \frac{10}{9} L I_{\parallel}^{DC,\exp} m_{\parallel}^{\exp}\sin(\omega t - \phi_{\parallel}^{\exp}). \tag{S3}$$

The total corrected reflected component is described by:

$$I_{\perp}^{corr} = I_{\perp}^{DC,corr} + I_{\perp}^{AC,corr}\sin(\omega t - \phi_{\perp}^{corr}). \tag{S4}$$

For convenience, we will define the following notation,

$$I_0 = I_{\perp}^{AC,corr}, \quad I_1 = 1.02 I_{\perp}^{DC,\exp} m_{\perp}^{\exp}, \quad I_2 = -\frac{10}{9} L I_{\parallel}^{DC,\exp} m_{\parallel}^{\exp}. \tag{S5}$$

Equation (S3) is simplified to

$$I_0 \cdot \sin(\omega t - \phi_{\perp}^{corr}) = I_1 \sin(\omega t - \phi_{\perp}^{\exp}) + I_2 \sin(\omega t - \phi_{\parallel}^{\exp}). \tag{S6}$$

Shifting the phase of each signal by $\phi_{\perp}^{\exp}$, and using $\Delta\phi^{\exp} = \phi_{\perp}^{\exp} - \phi_{\parallel}^{\exp}$, we get

$$I_0 \sin(\omega t + \phi_{\perp}^{\exp} - \phi_{\perp}^{corr}) = I_1 \sin(\omega t) + I_2 \sin(\omega t + \Delta\phi^{\exp}). \tag{S7}$$

After setting *ωt*=0, we obtain

$$\sin(\phi_{\perp}^{\exp} - \phi_{\perp}^{corr}) = \frac{I_2}{I_0}\sin(\Delta\phi^{\exp}), \tag{S8}$$

After setting *ωt*=π/2, we can obtain

$$\cos(\phi_{\perp}^{\exp} - \phi_{\perp}^{corr}) = \frac{I_1 + I_2}{I_0}\cos(\Delta\phi^{\exp}), \tag{S9}$$

By squaring Eq. (S8) and Eq. (S9) and using Pythagorean identity, we get

$$I_0 = \sqrt{(I_1)^2 + (I_2)^2 + 2I_1 I_2 \cos(\Delta\phi^{\exp})}, \tag{S10}$$

and the corrected phase is given by substituting Eq. (S10) in Eq. (S8):

$$\phi_{\perp}^{corr} = \sin^{-1}\left(\phi_{\perp}^{\exp} - \frac{I_2}{I_0}\sin(\Delta\phi^{\exp})\right). \tag{S11}$$

Appendix B

The Strickler-Berg formula is one of the most cited equations in the history of fluorescence that describes the fundamental relationship between the absorption spectrum ($\varepsilon(\bar{\nu})$) and the natural FLT (τ_n), meaning the FLT of a fluorophore in the absence of nonradiative processes.

In principle, the natural lifetime τ_n can be calculated by:

$$1/\tau_n = 2.88 \cdot 10^{-9} n^2 \left\langle \bar{\nu}_f^{-3} \right\rangle^{-1} \left(g_l / g_u \right) \int \frac{\varepsilon(\bar{\nu})\, d\bar{\nu}}{\bar{\nu}}, \tag{S12}$$

where n is the refractive index of the medium, $\left\langle \sim \bar{\nu}_f^{-3} \right\rangle^{-1} = \sim \bar{\nu}_f^{3}$ is the fluorescent maximum in cm^{-1}. g_l and g_u are the degeneracies of the lower and upper states, respectively. For fluorescence transition, the ratio is 1. $\varepsilon(\bar{\nu})$ is the molar absorptivity absorption spectra.

The Strickler-Berg formula is fundamental relation that applies to all fluorophores. However, this formula predicts the experimental FLT only for cases that follow several assumptions which are only often achieved in real systems. One of which is the absolute rigidity of the fluorophore in both the ground and excited state. An example to a rigid fluorophore is the fluorescein and thus, this equation can describe well the relation between the refractive index of the media and the

experimental FLT for fluorescein. However, generally interactions between the fluorophore and its solvent mask this behavior (since different quenching effects frequently dominate) and Eq. (S12) extremely overestimates the experimental FLT.

Appendix C

The histograms in the results section have normalized probability and they are characterized by the mode (the peak of each distribution) and the FWHM (the width of a distribution measured between the points on the y-axis which are half the maximum amplitude).

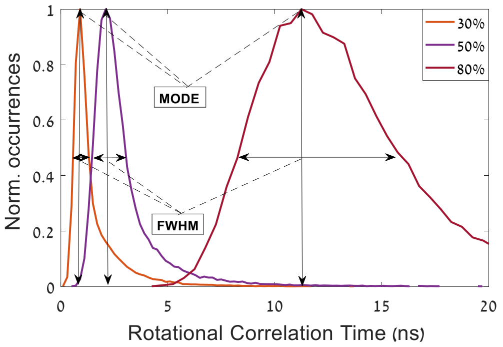

Fig. S1. The mode and FWHM of 3 θ distributions of Fluorescein-glycerol (Fl-Gly) systems with 3 different glycerol concentration (0%, 50% and 80%). It is evident that increasing the glycerol concentration increases both the mode and the FWHM.

For example, in Fig. S1, there are 3 different θ distributions of Fl-Gly system with 3 different glycerol concentration (30%, 50% and 80%). Clearly, the increase in glycerol concentration increases both the mode and the FWHM. Therefore, both the mode and the FWHM can imply on the increasing viscosity.

Appendix D

In order to confirm the attachment of each of the CDs to the AuNPs the morphologies and dimensions of the CDs and CDs-Au nanohybrid were investigated by TEM. The TEM images for the AuNPs alone found a size range from 14-18 nm (Fig. S2(a)). In addition, a prominent peak in the CDs FTIR spectra (Fig. S2(b)) at 1515 cm^{-1} indicated the existence of the -NH group, whereas

a broad peak at 1345 cm^{-1} indicated OH deformation vibrations. The change of the carbonyl peak of carboxylic acid from 1623 to 1609 cm^{-1} endorsing the establishment of an amide linkage confirmed the covalent attachment of AuNPs to the CD surface.

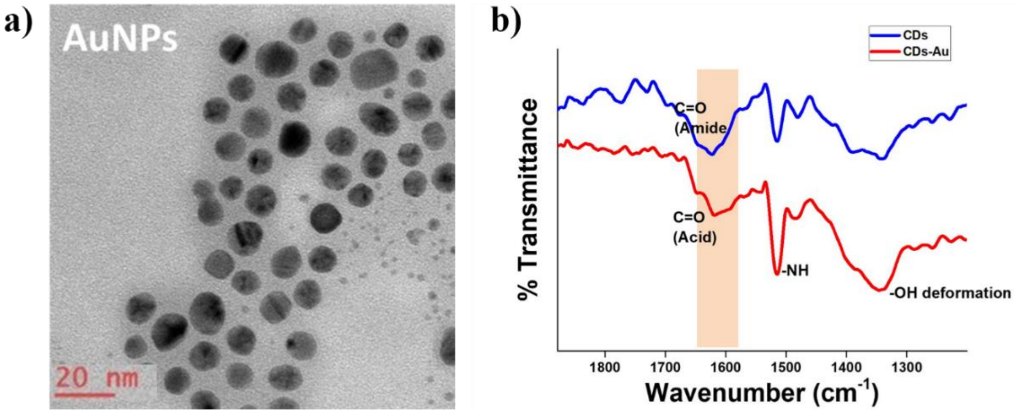

Fig. S2. TEM images of amine PEG coated AuNPs (**a**). FTIR spectra of CDs and CDs-Au (**b**). the prominent peak in the CDs at 1515 cm^{-1} indicated the existence of the -NH group, whereas a broad peak at 1345 cm^{-1} indicated OH deformation vibrations. The change of the carbonyl peak of carboxylic acid from 1623 to 1609 cm-1 endorsing the establishment of an amide linkage confirmed the covalent attachment of AuNPs to the CD surface.

Appendix E

Prior to using the polarized beam splitter in the emission path, we conducted a series of measurements with a linear polarizer, rotating the orientation angle of the polarizer (α) by 10 degrees with each measurement. By doing so, we were able to obtain polarization intensity data that followed a COS^2 pattern between the orientation angle and intensity, thereby validating the exact angle of both the parallel and perpendicular components (Fig. S3).

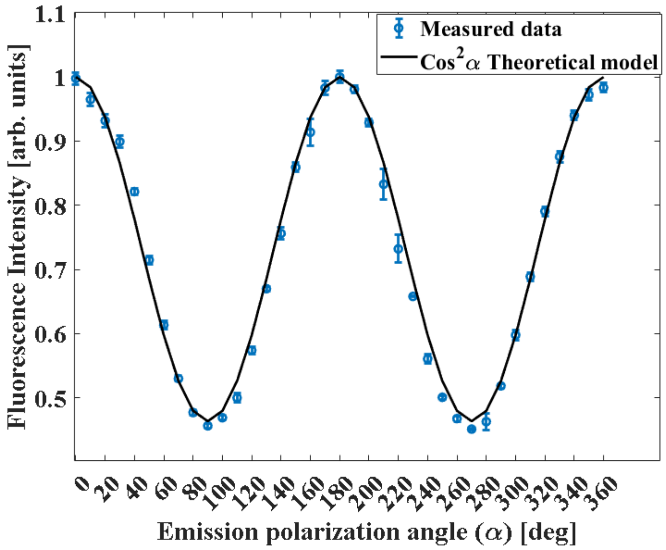

Fig. S3. The relation between the intensity and the orientation angle of the emission linear polarizer. A sequence of measurements was performed using a linear polarizer, where the orientation angle was altered by 10 degrees in each measurement. This approach enabled us to gather data on the polarization intensity that adhered to the expected COS^2 trend linking the angle of orientation to the intensity. Consequently, the precise angles for both the parallel and perpendicular components were confirmed.
